# *Mycolicibacterium brumae* is a Safe and Non-Toxic Immunomodulatory Agent for Cancer Treatment

**DOI:** 10.3390/vaccines8020198

**Published:** 2020-04-25

**Authors:** Marc Bach-Griera, Víctor Campo-Pérez, Sandra Barbosa, Sara Traserra, Sandra Guallar-Garrido, Laura Moya-Andérico, Paula Herrero-Abadía, Marina Luquin, Rosa Maria Rabanal, Eduard Torrents, Esther Julián

**Affiliations:** 1 Departament de Genètica i de Microbiologia, Facultat de Biociències, Universitat Autònoma de Barcelona, 08193 Barcelona, Spain; marc.bach@uab.cat (M.B.-G.); victor.campo@uab.cat (V.C.-P.); sandra.guallar@uab.cat (S.G.-G.); paula.herrero@uab.cat (P.H.-A.); marina.luquin@uab.cat (M.L.); 2 Bacterial Infections and Antimicrobial Therapies group, Institute for Bioengineering of Catalonia (IBEC), The Barcelona Institute of Science and Technology (BIST), 08028 Barcelona, Spain; lmoya@ibecbarcelona.eu (L.M.-A.); etorrents@ibecbarcelona.eu (E.T.); 3 Department of Cell Biology, Physiology and Immunology, Facultat de Veterinària, Universitat Autònoma de Barcelona, 08193 Barcelona, Spain; sandra.barbosa@uab.cat (S.B.); sara.traserra@uab.cat (S.T.); 4 Integrated Services of Laboratory Animals, Universitat Autònoma de Barcelona, 08193 Barcelona, Spain; 5 Unitat de Patologia Murina i Comparada, Departament de Medicina i Cirurgia Animals, Facultat de Veterinària, Universitat Autònoma de Barcelona, 08193 Barcelona, Spain; rosa.rabanal@uab.cat; 6 Microbiology Section, Department of Genetics, Microbiology and Statistics, Faculty of Biology, University of Barcelona, 643 Diagonal Ave., 08028 Barcelona, Spain

**Keywords:** bladder cancer, nontuberculous mycobacteria, BCG, safety, *Galleria mellonella*, mice

## Abstract

Intravesical *Mycobacterium bovis* Bacillus Calmette–Guérin (BCG) immunotherapy remains the gold-standard treatment for non-muscle-invasive bladder cancer patients, even though half of the patients develop adverse events to this therapy. On exploring BCG-alternative therapies, *Mycolicibacterium brumae*, a nontuberculous mycobacterium, has shown outstanding anti-tumor and immunomodulatory capabilities. As no infections due to *M. brumae* in humans, animals, or plants have been described, the safety and/or toxicity of this mycobacterium have not been previously addressed. In the present study, an analysis was made of *M. brumae*- and BCG-intravenously-infected severe combined immunodeficient (SCID) mice, *M. brumae*-intravesically-treated BALB/c mice, and intrahemacoelic-infected-*Galleria mellonella* larvae. Organs from infected mice and the hemolymph from larvae were processed to count bacterial burden. Blood samples from mice were also taken, and a wide range of hematological and biochemical parameters were analyzed. Finally, histopathological alterations in mouse tissues were evaluated. Our results demonstrate the safety and non-toxic profile of *M. brumae*. Differences were observed in the biochemical, hematological and histopathological analysis between *M. brumae* and BCG-infected mice, as well as survival curves rates and colony forming units (CFU) counts in both animal models. *M. brumae* constitutes a safe therapeutic biological agent, overcoming the safety and toxicity disadvantages presented by BCG in both mice and *G. mellonella* animal models.

## 1. Introduction

Bladder cancer (BC) currently represents the second most common malignancy of the urinary tract [1]. In 2018, approximately 549,393 newly diagnosed cases and 199,922 deaths from bladder cancer were reported worldwide [2]. Most BC cases are diagnosed when patients present with macroscopic hematuria, and confirmed after transurethral resection of bladder tumor (TURBT) [3]. In approximately 75% of BC patients, the disease is localized in the mucosa layer (Ta or carcinoma in situ [CIS]) or submucosa layer (T1) [1,4]. In these initial stages, known as non-muscle-invasive BC (NMIBC), intra-vesical/intra-bladder (IB) instillations of live *Mycobacterium bovis* Bacillus Calmette–Guérin (BCG) are still the gold standard treatment to avoid recurrence and progression of the disease [3,5].

BCG comprises a family of related sub-strains derived from a virulent strain of *M. bovis*, the causative agent of bovine tuberculosis. BCG was generated after eleven years of continuous culture passages, demonstrating, first in animal models and later in humans, in 1921, that it conferred protection against tuberculosis infection [6,7]. In the mid-1970s, Morales and his colleagues reported the efficacy of BCG in the control of recurrence and progression of NMIBC [8], and after several clinical trials, BCG was finally approved by the Food and Drug Administration (FDA) (in 1990) for the treatment of NMIBC [9,10,11]. The mechanism of action of BCG, as well as the best practice management, is still not completely understood. Nevertheless, the data support that the anti-tumor effects of BCG are due to an inhibition of tumor proliferation through infection of urothelial cells, a powerful local immune response involving the activity of neutrophils, macrophages, and dendritic cells, as well as the release of different cytokines such as Interleukin (IL)-1, IL-2, IL-6 and IL-8, Tumor Necrosis Factor (TNF)-α, and Interferon (IFN)-γ [1,12,13].

Despite its demonstrated efficacy, BCG treatment presents with local adverse effects in more than 50% of patients, systemic adverse effects in around 30% of patients, and 1% to 5% of patients suffer rare but severe complications such as pulmonary infections or sepsis [14,15,16,17]. In fact, after transurethral resection of the tumor, there must be a delay of around two weeks before patients can proceed to IB BCG instillations, in order to avoid the systemic dissemination of the bacilli through the wound. Nevertheless, the high number of instillations during the induction and maintenance treatment (up to three years) assume a risk of infection whenever traumatic instillation occurs.

In order to avoid the possibility of BCG infection, different strategies involving non-pathogenic bacteria different from mycobacteria, or mycobacteria-derived agents, are currently under investigation [18,19,20,21]. The majority of these strategies consider the use of heat-killed or γ-irradiated mycobacteria, or fractions of mycobacteria cells. However, different studies have highlighted the importance of the use of live mycobacteria (at least at the first instillation) to achieve an adequate response against bladder tumors [22,23]. An interesting option to substituting BCG could be the use of non-infectious mycobacteria. Studies from our laboratory using this alternative demonstrated the anti-tumor and immunostimulatory capacity of *Mycolicibacterium brumae* (basonym *Mycobacterium brumae*) [Appendix A], a saprophytic mycobacterium with a rapid growth rate in vitro. *M. brumae* inhibits BC cell proliferation in vitro, triggers a cytotoxic profile in stimulated peripheral mononuclear blood cells against BC cells ex vivo, and increases survival of tumor-bearing mice by triggering both an IB and a systemic immune response that leads to tumor clearance [5,24,25].

Until now, no infections due to *M. brumae* in humans, other animals, or plants have been described. Both bladder tumor cells and macrophages can eliminate viable *M. brumae* in a few days after infection whereas BCG remains viable inside the cells [24,25]. Moreover, no colony forming units (CFU) have been isolated in tumor-bearing mice treated intravesically with *M. brumae*, suggesting the lack of pathogenicity of this species. However, no safety or toxicity studies have been undertaken. In this study, immunosuppressed and immunocompetent mice, after intravenous (IV) and IB administration, as well as the *Galleria mellonella* model, were used to assess the safety and potential toxicity of live and γ-irradiated *M. brumae.*

## 2. Materials and Methods

### 2.1. Bacterial Cultures

*M. bovis* BCG Connaught (ATCC 35745) and *M. brumae* (ATCC 51384^T^) were grown on Middlebrook 7H10 medium (Difco Laboratories, Detroit, Michigan) supplemented with 10% Oleic-Acid-Dextrose-Catalase (OADC) enrichment for 3 or 1 week, respectively, at 37 ℃. BCG and *M. brumae* cells were resuspended in sterile phosphate buffered saline (PBS) and adjusted to a 1.0 McFarland turbidity standard as previously described [26] in order to get the required concentration for each treatment. To confirm the dose of each experiment, representative bacterial suspensions of each treatment were serially diluted in PBS, and CFU were counted after plating on Middlebrook 7H10 medium as previously described [27]. Irradiation of *M. brumae* for intravenous infection and intravesical treatment was performed at Aragogamma S.L. as previously described [5].

### 2.2. Intravenous (IV) and Intravesical (IB) Mice Treatments

Animal experiments were performed according to procedures approved by the Ethics Committee on Animal Experiments (CEEA) of the *Universitat Autònoma de Barcelona* (UAB) and the Commission of Animal Experimentation of *Generalitat de Catalunya* (UAB procedure numbers: 3632 and 3633; and *Generalitat de Catalunya* procedure number: 9528). The minimum number of animals was used in compliance with current regulations. The welfare of the animals was considered in terms of number and extent of procedures to be performed. Before starting the experiment, mice were randomized according to their weight. The animals were weighed and evaluated on a daily basis for their general appearance, clinical signs, and behavior, and were euthanized in order to avoid unnecessary suffering.

**Figure 1:**
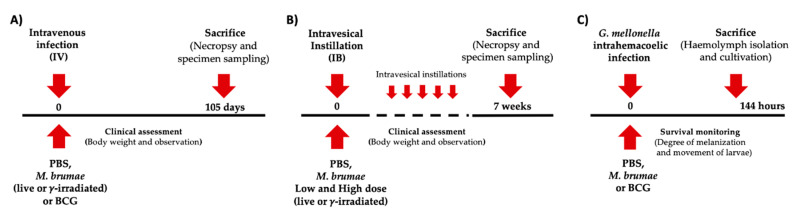
Schematic schedule of (**A**) intravenous infection in severe combined immunodeficient (SCID) mice, (**B**) intravesical treatments in BALB/c, and (**C**) intrahemacoelic infection in *G. mellonella* larvae. Abbreviations: PBS, phosphate buffered saline; BCG, Bacillus Calmette–Guérin.

The IV infection was carried out in 8-week-old C.B-17/IcrHan®Hsd-Prkdc^scid^ (severe combined immunodeficient (SCID) mice) (Envigo) (8 female mice/group) [28,29]. Mice were IV treated via the lateral tail vein with a dose of 2 × 10^6^ mycobacteria CFU of live *M. brumae*, γ-irradiated *M. brumae*, or live BCG in 0.1 mL of PBS, or 0.1 mL of PBS as a control. At 15 weeks, the surviving mice were sacrificed by exsanguination under terminal inhalation anesthesia for a complete systematic necropsy and histopathology assessment. Collected blood was processed for biochemistry and hematology parameters, and the main mycobacteria-target organs (liver, lung and spleen) were removed for quantitation of CFU and/or histopathology analysis (schedule is shown in Figure 1A).

The evaluation of safety and toxicity of *M. brumae* after repeated IB administrations was performed in 8-week-old BALB/c OlaHsd (Envigo) mice (8 female mice/group) [25,30]. A dose of 2 × 10^6^ CFU/mouse (low dose) or 2 × 10^9^ CFU (high dose) of live or γ-irradiated *M. brumae* in 0.1 mL PBS, or PBS alone for control group, was administered through IB instillations (dwell time 60 minutes) [5]. Animals were treated weekly for 6 weeks (Figure 1B), and were euthanized two weeks after the last treatment. A complete systematic necropsy was undertaken. Lung, spleen, liver, and kidney were aseptically removed to quantify CFU. Urinary bladders were also preserved for histopathology analysis.

### 2.3. G. mellonella Infection Model

*G. mellonella* larvae were reared at 34 ºC and fed an artificial diet consisting of corn flour, wheat flour, powdered milk, cereals, brewer’s yeast, honey, and glycerol. Larvae were infected through an intrahemacoelic injection with a Hamilton 22-gauge syringe using three different concentrations of *M. brumae* and BCG corresponding to 10^4^, 10^5^, and 10^6^ CFU/larva in 0.01 mL of PBS prepared as described above. Fifteen larvae were used for each group, and the experiment was performed four times (n = 60). A group of fifteen larvae injected with PBS was also included in each experiment as a control group (Figure 1C).

### 2.4. Mycobacteria CFU Counting in Different Organs from Mice and G. mellonella Hemolymph

Lungs, spleens, and livers from IV-treated mice were weighed and imaged after collection. A portion of spleen, liver, and upper right lung lobe from each mouse was then weighed and processed for CFU quantification. Furthermore, kidneys from mice subjected to IB instillations were also analyzed, since in the context of IB instillations, kidneys are another critical organ due to its direct contact to the bladder lumen. Lungs and livers were resuspended in 1mL of PBS and disrupted using glass homogenizers, while spleens were resuspended in 5 mL of Roswell Park Memorial Institute medium (RPMI, Lonza, Switzerland), and disrupted using forceps and by passing through a needle. Afterwards, serial dilutions from the homogenates were cultured on 7H10 agar plates. Plates were incubated at 37 °C for 1 week or 3 weeks for *M. brumae* or BCG, respectively, in order to count CFU. In addition, hemolymph from surviving mycobacteria-treated larvae at 144 h post-infection was collected and cultivated on 7H10 agar plates in order to count CFU. Data reported represent the mean ± SD of CFU/g for each organ and CFU/mL from hemolymph of larvae.

### 2.5. Determination of Biochemical and Hematological Parameters

Blood samples were obtained through a cardiac puncture from IV-infected and IB-inoculated mice. Blood was divided into two different tubes: an EDTA-containing tube for hematology analysis and a lithium heparin-containing tube for the biochemical analysis. Plasma was obtained from the blood of lithium heparin tubes by centrifugation (15 min, 3500 rpm/1200× *g*, 4 °C, Heraeus Megafuge 11R) and stored at −80 °C until biochemical analysis. A broad range of biochemical and hematology parameters were analyzed using Olympus AU400 chemistry and ADVIA 120 (Siemens®) hematology analyzers, respectively. Biochemistry analysis included the quantification of alanine transaminase (ALT), aspartate transaminase (AST), creatinine, iron, alkaline phosphatase, glucose, potassium, total proteins, sodium, urea, and albumin. For the hematology analysis, whole blood was immediately processed for the white blood cell counts (WBCB), red blood cell counts (RBC), hematocrit, platelets, total neutrophils, total lymphocytes, total basophils, total eosinophils, and total monocytes.

### 2.6. Histopathological Analysis in Mouse Organs

At necropsy, the representative tissues of all systems from IV-infected mice and urinary bladders from IB-inoculated mice were immediately fixed in 4% formaldehyde and embedded in paraffin blocks. Hematoxylin and eosin staining was performed on 2–3 μm sections of all specimens. Histopathology assessment included the analysis of stomach, duodenum, pancreas, jejunum, ileum, cecum, colon, lymph nodes, liver, spleen, ovary, uterus, vagina, kidneys, adrenal glands, urinary bladder, salivary glands, submandibular lymph nodes, esophagus, trachea, lung, heart, thymus, brain, sciatic nerve, skin, muscle, aorta, and bone marrow in IV-infected mice, and the urinary bladders of IB-inoculated mice. Representative images were taken from all processed specimens. Histological damage was scored as follows: 0, no apparent lesions; 1, very slight lesions; 2, mild lesions; 3, moderate lesions; 4, intense lesions.

### 2.7. Statistical Analysis

The statistical significance of CFU counts and histopathology analysis (organs from IV-infected mice and urinary bladders from IB-inoculated mice) was assessed using the non-parametric one-way analysis of variance by ranks Kruskal–Wallis H test. Mean values ± standard deviation (SD) of weight gain and weight loss were compared between different treatment groups using two-way ANOVA. Body weight, weight of organs, hematology, and biochemistry results were expressed as mean ± SD and were compared using one-way ANOVA. Mouse and *G. mellonella* survival was analyzed using Log–Rank (Mantel–Cox) test and plotted as Kaplan–Meier curves. Significance was considered as *p* < 0.05. All statistical analysis and graphics were performed using GraphPad Prism (version 6.0c for macOS; GraphPad Software, San Diego, CA, USA).

## 3. Results

### 3.1. M. brumae-Treated Animals Showed 100% Survival Rates after Treatments

**Figure 2:**
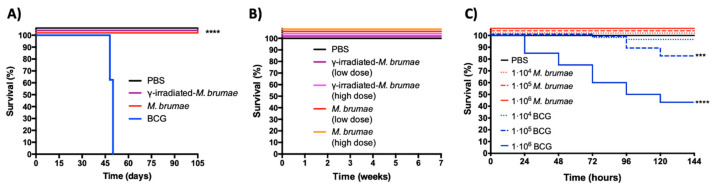
Kaplan–Meier survival curves after intravenous (IV)-infection and intravesical (IB)-inoculation in mice and intrahemacoelic infection in *G. mellonella*. (**A**) SCID mice (n = 8/group) received a single intravenous infection of live (red) or (purple) γ-irradiated *M. brumae* or Bacillus Calmette–Guérin (BCG) (blue), or PBS (black). BCG-infected mice survived for over 48 days, whereas the rest of mice groups survived until the end of the study. **** *p* < 0.0001 (Mantel–Cox test). (**B**) Balb/C mice (n = 8/group) received IB instillations with low (purple) or high doses (violet) of live γ-irradiated; low (red) or high doses (orange) of live *M. brumae;* or PBS (green). All animals survived until the end of the experiment; (**C**) *G. mellonella* larvae (n = 60/group) were infected with 1 × 10^4^; 1 × 10^5^ or 1 × 10^6^ CFU/larvae of *M. brumae* or BCG, or PBS as control *** *p* < 0.0005; **** *p* < 0.0001 (Mantel–Cox test).

Three different animal models were assayed in order to evaluate the safety and toxicity of *M. brumae*. In the first model of IV infection in SCID mice, all animals treated using γ-irradiated or live *M. brumae* survived until the end of the study as shown in Figure 2A. Mice infected with live *M. brumae* showed a statistically significant body weight increase compared to PBS-treated mice from week 8 after infection to almost the end of the study (Figure S1A). At day 84, for instance, live *M. brumae*-infected mice showed an increase of percentage weight with respect to their initial weight of 40.73 ± 17.75, while PBS treated mice showed an increase of 30.64 ± 8.70 (*p* < 0.05). On the other hand, BCG-infected mice started losing weight (Supplementary Figure S1A), as well as presenting with clinical signs from the fourth week after infection. Humane endpoints were applied in all mice from the BCG group between 48–55 days after infection (Figure 2A).

In the case of mice IB treated with both γ-irradiated and live-*M. brumae*, no clinical signs or weight decreases were observed throughout the experiment, even in animals treated with the highest doses (Supplemental Figure S1B). All animals showed an increase in body weight at the end of the experiment, compared to those recorded at the beginning of the experiment, with no differences between *M. brumae*-treated groups and the PBS-treated group (Figure S1). All animals survived until the end of the experiment (Figure 2B).

When *M. brumae* was injected into *G. mellonella*, larvae survived until the end of the experiments (six days after injection) regardless of the concentration injected, which is significant when compared to survival rates of larvae infected with BCG at 10^5^ and 10^6^ CFU/larvae (Figure 2C, *** *p* < 0.0005; **** *p* < 0.0001). At the highest mycobacteria concentration, survival rates in *M. brumae*-infected larvae reached 100%, while in BCG-infected larvae it decreased by up to 43%.

### 3.2. No CFU Were Recovered from M. brumae-Treated Animals at the End of the Experiments

**Figure 3:**
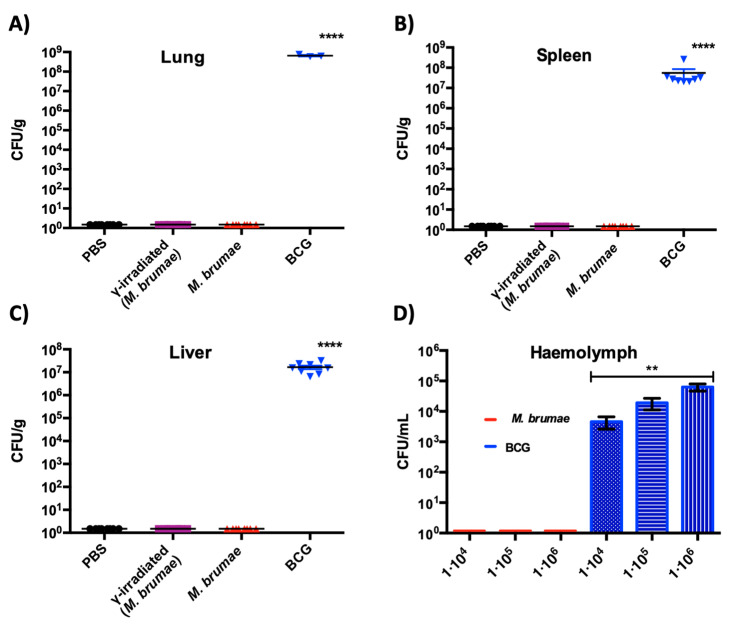
Colony forming units (CFU) counts obtained from (**A**) lungs, (**B**) spleens, and (**C**) livers from mycobacteria-infected SCID mice. No CFUs were detected in the organs of *M. brumae*-infected mice, while CFU counts were significantly elevated in the lung, spleen and liver tissues of BCG-infected mice. (**D**) Bacterial burden recovered in hemolymph samples from *G. mellonella* larvae. While no CFU counts were observed in *M. brumae*-infected larvae, high CFU counts were observed in BCG-infected larvae. Data are presented as the mean values ± SD of the mycobacteria CFU counts. ** *p* < 0.01, **** *p* < 0.0001 (Kruskal–Wallis H test).

While CFU counts were obtained in cultures of lungs, spleen, or livers from IV BCG-infected SCID mice, no CFU were obtained in organs extracted from IV *M. brumae*-infected animals (*p* < 0.0001) (Figure 3A-C). Both lungs and spleens from IV BCG-infected mice showed weight differences compared to IV PBS-treated mice (*p* < 0.001). No significant differences were observed in the weights of livers between IV-treated groups (Figure S1C). With regards to the organs from IB-treated mice, no *M. brumae* CFU were detected, and no differences were observed in the weights of any organ in any experimental group (Figure S2). Finally, no CFU were observed in hemolymph cultures from 10^4^, 10^5^, or 10^6^ *M. brumae*-infected *G. mellonella* larvae 144 hours after infection. In contrast, up to 10^3^–10^4^ CFU/mL were obtained in hemolymph cultures from BCG-infected larvae (Figure 3D).

### 3.3. IV and IB M. brumae Treatments Did not Alter Biochemical and Hematology Parameters Compared to Control Mice

To assess the toxicity of *M. brumae*, a wide range of biochemical and hematology parameters was analyzed in the blood collected from the treated animals.

In IV infection experiments, AST, ALT, potassium and urea values were significantly higher in the IV BCG-infected group compared with the rest of the groups. Sodium was slightly increased compared with γ-irradiated *M. brumae* group. Furthermore, creatinine, iron, and glucose values in the IV BCG-infected group were significantly decreased compared with those obtained in the rest of the groups. No differences were observed in alkaline phosphatase values between groups (Table 1). Similarly, differences in hematology parameters were only found in IV BCG-infected mice. WBC counts were increased in IV BCG-infected mice, with neutrophils and lymphocytes the cells showing the highest increases, followed by basophils, and lastly, monocytes and eosinophils (Table 2). No differences were observed between *M. brumae*-infected groups and control mice in biochemical or hematology analyses.

With regards to IB-inoculated mice, no significant differences were observed in the biochemistry or hematology parameters between groups, compared to control mice, even when high doses of live *M. brumae* were instilled into the bladder (Table 3 and Table 4).

### 3.4. Histopathology Analysis Reveals the Safety and Lack of Toxicity of M. brumae Treatments in Mice

**Figure 4:**
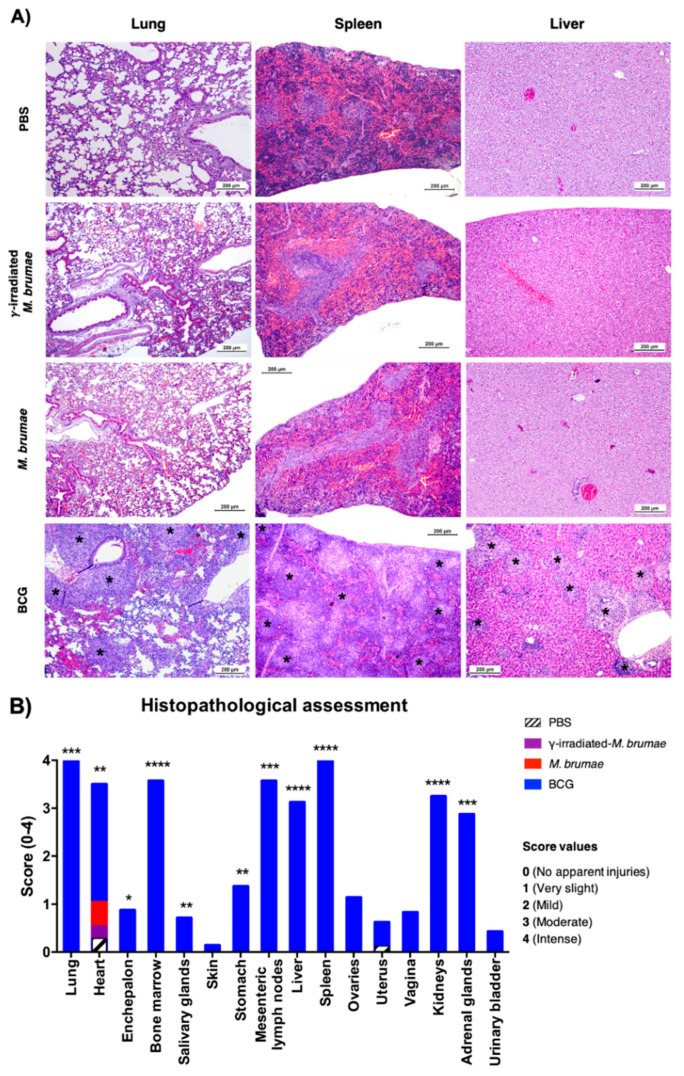
Histopathology analysis of the different organs from IV-infected mice. (**A**) Hematoxylin and eosin staining of lungs, spleens and livers from PBS, γ-irradiated-*M. brumae*, *M. brumae* and BCG- IV infected mice. Only BCG-infected mice showed lesions consisting of granulomas with inflammatory component (*, black asterisks). The lesions were massive, showing in some organs a coalescent pattern (note lung and spleen sections). (**B**) A score (0 to 4) in accordance with the degree of the histological findings. Only BCG-infected mice exhibited systemic injuries of maximum gravity. Statistically significant differences were seen in 11 out of 16 organs between BCG-infected mice and the other infected mice. * *p* < 0.05; ** *p* < 0.01; *** *p* < 0.001, and **** *p* < 0.0001 (Kruskal–Wallis H test).

Histological damage scoring was performed to assess the grade of tissue lesions in association with the systemic mycobacterial infection. Statistically significant differences (*p* < 0.05) were observed in several analyzed tissues in IV BCG-infected mice compared to *M. brumae-*infected or control groups. Lesions compatible with high severity systemic multi-organic infection were observed, as shown in Figure 4A. Lesions were described as coalescent granulomas of parenchymal distribution with an inflammatory component composed of macrophages, epithelioid cells, and to a lesser extent, neutrophils. Statistically significant differences were seen in the stomach, mesenteric lymph nodes, liver, kidneys, adrenal glands, heart, bone marrow, spleen, lungs, salivary glands, and encephalon between the BCG-treated group and the other groups (live *M. brumae*, γ-irradiated *M. brumae* and PBS) (Figure 4B). No differences were observed in duodenum, jejunum, ileum, colon, caecum, pancreas, ovaries, uterus, vagina, urinary bladder, esophagus, trachea, thymus, nerve, skin, muscle, and aorta-cava between groups. Some animals from both *M. brumae*-infected and PBS groups showed epicardial mineralization with no pathological or clinical relevance (Figure 4B).

**Figure 5:**
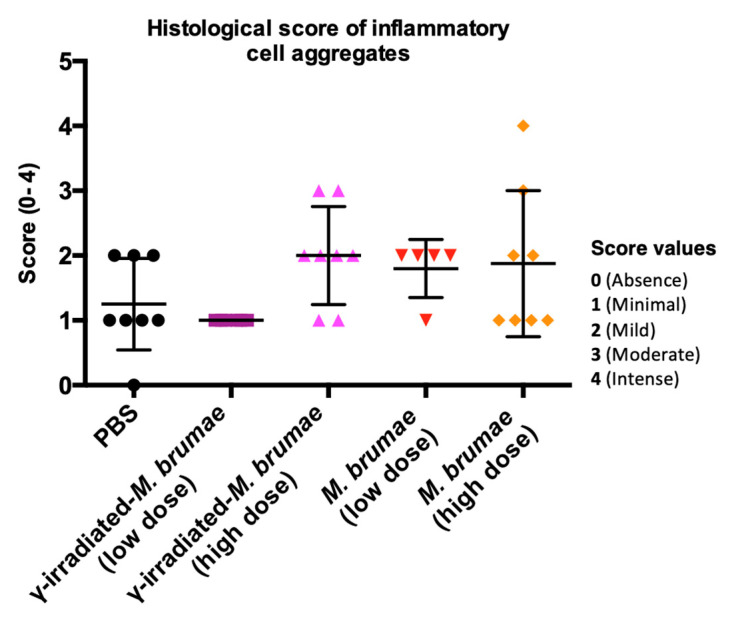
Histology score in bladders from IB-treated mice. A score (0 to 4) in accordance with the degree of inflammatory cells observed during the histopathology analysis of the bladders.

Histopathology analysis of urinary bladders revealed inflammatory cell aggregates in the lamina propria of some bladders in all IB-treated experimental groups. For this reason, it is not considered a histological finding related to the experimental treatments. Nevertheless, a score from 0 to 4 was assigned for each animal based on the degree of inflammatory cells observed (Figure 5). Although no significant differences were observed between groups, the high dose of γ-irradiated *M. brumae* group was the condition with the highest inflammatory score followed by the high dose of live *M. brumae* group. On the contrary, the group with the lowest inflammatory score was the γ-irradiated *M. brumae* low dose group. Only one animal achieved a histology score of 4, showing chronic cystitis that also affected the muscular layer (Figure 5), which can be associated with repeated intravesical administrations.

## 4. Discussion

Non-tuberculous mycobacteria, such as *M. brumae,* are an extremely common species found in the environment, in both soil and water sources. The vast majority are innocuous for animals and plants, but some of them can cause opportunistic diseases, such as *Mycobacterium abscessus*, *Mycobacterium avium,* or *Mycobacterium fortuitum,* among others [31]. These cases of infection occur mainly in immunocompromised hosts, but can also be produced in immunocompetent hosts. The extraordinary immunomodulatory potential of mycobacteria led to some of these non-tuberculous species being used for the treatment of infectious diseases and cancers. Among them, *M. brumae* has been described as a potential immunotherapeutic agent for NMIBC treatment. *M. brumae* shares the anti-tumor ability of BCG, as well as its capacity to trigger an immune response during intravesical instillations [5,24,25,32]. In contrast to BCG, no CFU have been isolated in *M. brumae*-treated tumor bearing mice [24,25], but a systematic safety and toxicity study on *M. brumae* needs to be performed.

Our results in IV infection experiments demonstrated that *M. brumae* is a safe agent, even in immunodeficient animals. No pathological findings, such as clinical signs, weight loss, alterations in biochemical or hematological blood parameters, necropsy findings, or in the histological assessment, were found in IV *M. brumae*-infected mice. Moreover, no infection was observed in any of the studied organs. No CFU counts were found in the main mycobacteria target organs: liver, lung, and spleen. In contrast, as expected [33,34,35,36,37], SCID mice infected with BCG did not reach the endpoint of the study (105 days). Animals of the BCG group presented with clinical and pathological findings compatible with a severe systemic and multi-organ infection.

Histopathology findings, together with alterations in several blood parameters analyzed in the BCG treatment group may be related to the failure of the most important organs. BCG infection resulted in the formation of liver granulomas mainly composed of Kupffer cells and blood monocytes. According to reported data, these cells limit the dissemination of mycobacteria by ensuring their phagocytosis [38], and increasing the production of AST and ALT (enzyme indicators of liver injury) [39,40], as we found in our experiments (Figure 4A and Table 1). Histopathology analysis of lungs and spleens also revealed the presence of granulomas (Figure 4A). Our results are in agreement with other studies using the same BCG sub-strain (Connaught), showing granulomas in all three abovementioned organs [41]. Some other studies in which SCID mice were intravenously infected with the BCG Pasteur sub-strain described the formation of granulomas only in liver and spleen, but not in lungs, attributing this fact to a characteristic deficiency of CD4+ T-cell-mediated immunity in this immunodeficient mouse model [42].

Biochemical and hematology analysis also confirmed a classical systemic BCG infection. In addition to the previously mentioned AST and ALT liver enzymes, higher blood levels of potassium and sodium were also found in IV BCG-infected mice compared with other experimental groups. Pathological findings in adrenal glands and kidneys, which regulate potassium excretion [43,44], might explain a higher potassium level in blood. In addition, high and low values of urea and creatinine, respectively, together with the observed kidney lesions might be attributable to kidney failure. As expected, low blood glucose and iron levels were found in BCG-infected animals, all of which showed clinical signs of systemic illness. Furthermore, as commonly seen in mycobacterial infections, iron levels may also decrease due to different strategies by mycobacteria to remove iron from the host [45,46]. Likewise, the hematology parameters indicated an inflammatory response due to the BCG infection, with increased WBC and RBC, which was absent in mice infected with live and γ-irradiated *M. brumae*. The total neutrophil count in BCG-treated mice suggested a classic neutrophilia typical of the advanced stages of tuberculosis, which could act as a Trojan horse harboring bacilli and promoting then the growth of *Mycobacterium tuberculosis* in the SCID mice [47,48].

Overall, IV BCG infection resulted in 100 % mortality, with survival times between days 48 and 55 of the study, which is in agreement with previous studies using different BCG sub-strains [28,49,50]. Clinical symptoms started by day 33 of the study and progressed until euthanasia was used as a humane endpoint.

Our results in the *G. mellonella* model also confirmed the safety of *M. brumae* compared to BCG infection. This model is largely being studied for the assessment of drug efficacy, dosing, and toxicity of different bacterial, viral, and fungal pathogens, due to the few technical requirements. Recent studies have demonstrated the validity of this model in the study of mycobacteria infections and to evaluate anti-mycobacterial drugs [51].The pathogenicity of different mycobacteria species could be reflected in the survival rates of *G. mellonella* after infection [52]. Survival of larvae and CFU counts in hemolymph demonstrated that *M. brumae* is eliminated from the organism within 144 hours after infection, while BCG survives inside the larvae, additionally decreasing larval survival rates. Therefore, this animal model also corroborates the safety of *M. brumae* infection.

The safety and toxicity assessment of *M. brumae* in BALB/c mice following repeated intravesical instillations with low and high doses of live and γ-irradiated *M. brumae*, did not show any significant differences in hematology or biochemistry parameters, or in urinary bladder histopathology between groups. These results are in agreement with our previous studies in the orthotopic murine model of bladder cancer, in which we did not observe granulomatous infiltration typical of pathogenic mycobacteria infection in animals treated with *M. brumae*. It is worth mentioning that four weekly instillations is sufficient to inhibit tumor growth and induce an adequate immune response in this orthotopic murine model of the disease [5,53]. We have previously demonstrated that after the first instillation in an orthotopic murine model using live *M. brumae*, further sequential instillations using γ-irradiated *M. brumae* maintain a statistically significant survival rate compared to non-treated tumor-bearing mice, although there are no statistically significant differences between survival rates of live-*M. brumae*-treated tumor-bearing mice and γ-irradiated *M. brumae*-treated mice [5]. For this reason, we considered it relevant to study the toxicity of live and γ-irradiated *M. brumae*. The high dose used in the present study, and the length of the treatment (6 weeks) corresponds to the usual administration in NMIBC patients in order to avoid recurrences and progression of the disease. The absence of clinical signs and bacterial burden in mice and in the processed organs also corroborate the safety and non-toxicity of *M. brumae* after IB instillation.

We cannot rule out a transient change in some hematological and/or biochemical parameters soon after IV or IB infection using live or γ-irradiated *M. brumae*. In our study, all parameters were only analyzed after finishing the experiment, and we did not include animal groups to be sacrificed at different time-points, due to ethical reasons. Our experiments were designed in the context of the use of *M. brumae* for NMIBC treatment. Thus, our main aim was to detect any local or systemic adverse events after the intravesical instillation of an overdose of *M. brumae,* and after an IV infection of *M. brumae*, since the worst adverse event in the treatment of NMIBC patients is the risk of systemic infection after traumatic intravesical instillations. Therefore, our experiments demonstrated that *M. brumae* is a safe agent, even in immunodeficient animals. The present results, together with the previously described immunomodulatory effect of *M. brumae* in tumor-bearing mice, leads us to propose the use of this mycobacterium for the treatment of other diseases in which the immune response is a key factor for their cure. In the context of cancer treatment, this safe agent could even be included as an adjuvant therapy together with other therapeutic options such as cancer vaccines currently under development [54,55].

## 5. Conclusions

We can conclude that both γ-irradiated and live *M. brumae* is innocuous when compared to *M. bovis* BCG exposure under the experimental conditions of the present study. Biochemical and hematology parameters in blood and histopathological analysis in a wide range of organs were similar in all *M. brumae* IV-infected or IB-inoculated mice compared to mice treated with the vehicle. Moreover, no *M. brumae* bacilli were found in mouse organs at the end of the experiments. Thus, these results, together with the previously reported data, reinforce the premise that *M. brumae* is a safe and non-toxic biological agent for use in intravesical treatment of NMIBC.

## Figures and Tables

**Table 1:** Assessment of biochemical parameters in IV-infected SCID mice.

Treatment	PBS		γ-Irradiated *M. brumae*		*M. brumae*		BCG		*p* Value
Parameters	Mean ± SD	Range	Mean ± SD	Range	Mean ± SD	Range	Mean ± SD	Range	
**ALT** (U/L) ^a^	17.86 ± 5.82	(11.30–30.80)	17.89 ± 6.39 ^1^	(12.20–31.50)	16.74 ± 3.05 ^1^	(14.00–22.60)	61.53 ± 36.81 ^3^	(27.80–100.80)	< 0.001
**AST** (U/L) ^b^	73.50 ± 38.43	(42.00–163.00)	51.29 ± 10.81 ^1^	(42.00–74.00)	65.86 ± 17.30 ^1^	(49.00–88.00)	326.00 ± 74.62 ^4^	(249.00–428.00)	< 0.0001
**Creatinine** (mg/dL)	0.27 ± 0.01	(0.25–0.29)	0.27 ± 0.02	(0.24–0.28)	0.28 ± 0.01 ^1^	(0.27–0.30)	0.23 ± 0.04 ^5^	(0.17–0.28)	< 0.05, < 0.01 *
**Iron** (µg/dL)	224.11 ± 40.55	(158.40–274.10)	218.26 ± 43.96	(156.40–276.70)	203.37 ± 9.89 ^2^	(188.10–214.50)	119.84 ± 31.02^5^	(88.00–157.70)	< 0.01 ^#^, < 0.001
**Alkaline Phosphatase** (mmol/L)	72.54 ± 4.71	(67.20–78.46)	77.69 ± 11.24	(68.17–100.46)	80.10 ± 7.99 ^1^	(66.90–90.02)	76.70 ± 11.75 ^6^	(68.39–85.00)	ns
**Glucose** (mg/mL)	271.98 ± 26.89	(232.20–317.40)	285.51 ± 45.10	(233.70–352.00)	269.60 ± 44.31 ^1^	(213.30–340.10)	121.37 ± 3.55 ^3^	(118.20–125.20)	< 0.0001
**Potassium** (mmol/L)	4.64 ± 0.74	(3.61–5.90)	4.89 ± 0.70	(3.96–5.75)	4.27 ± 0.55	(3.65–5.10)	5.95 ± 0.66 ^5^	(5.35–6.84)	< 0.05, < 0.01 ^&^, < 0.0001 ^
**Sodium** (mmol/L)	147.00 ± 1.61	(143.70–148.80)	146.33 ± 1.32	(144.50–148.10)	148.88 ± 1.64	(146.50–151.10)	149.14 ± 1.86 ^5^	(147.00–151.20)	< 0.05
**Urea** (mg/dL)	47.01 ± 7.83	(33.10–53.60)	44.21 ± 9.36	(34.20–60.50)	44.87 ± 8.61 ^1^	(35.50–60.60)	68.65 ± 16.27 ^4^	(55.60–92.20)	< 0.01
**Albumin** (g/dL)	2.87 ± 0.12	(2.72–3.10)	2.82 ± 0.09	(2.61–2.89)	2.82 ± 0.10 ^1^	(2.71–3.00)	2.14 ± 0 ^7^	−	ns
**Total Protein** (g/dL)	4.88 ± 0.19	(4.57–5.20)	4.77 ± 0.18	(4.38–4.93)	4.84 ± 0.12 ^1^	(4.69–5.04)	4.66 ± 0 ^7^	−	ns

Table shows mean and standard deviation (± SD) of different biochemical parameters in PBS-, γ-irradiated *M. brumae-*, *M. brumae-* and BCG-IV-infected mice. Abbreviations: ^a^ ALT (Alanine transaminase); ^b^ AST (Aspartate transaminase). Data are expressed as mean ± SD and the range of each parameter for each experimental group. N = 8 except for indicated parameters of experimental groups that had insufficient volumes of sample: ^1^ n = 7; ^2^ n = 6; ^3^ n = 3; ^4^ n = 4; ^5^ n = 5; ^6^ n = 2 and ^7^ n = 1). Significant differences were established by one-way ANOVA comparing the experimental groups versus BCG except: * (*M. brumae* vs. BCG), ^#^ (*M. brumae* vs. BCG), ^&^ (PBS vs. BCG) and ^ (*M. brumae* vs. BCG).

**Table 2:** Assessment of hematology parameters in IV-infected SCID mice.

Treatment	PBS		γ-Irradiated *M. brumae*		*M. brumae* ^1^		BCG ^1^		*p* Value
Parameters	Mean ± SD	Range	Mean ± SD	Range	Mean ± SD	Range	Mean ± SD	Range	
**WBCB**^a^ (×10^3^ cells/µL)	0.84 ± 0.23	(0.46–1.06)	1.14 ± 0.67	(0.65–2.55)	0.71 ± 0.23	(0.50–1.02)	11.40 ± 5.13	(5.00–18.14)	< 0.001
**RCB**^b^ (×10^3^ cells/µL)	9.35 ± 0.47	(8.88–10.23)	9.84 ± 0.27	(9.40–10.25)	9.29 ± 0.29	(8.97–9.75)	12.17 ± 0.57	(11.41–12.80)	< 0.001
**Hematocrit** (%)	40.16 ± 1.84	(37.80–42.20)	41.67 ± 1.08	(40.00–43.20)	40.28 ± 1.06	(38.9–41.9)	42.38 ± 2.10	(40.00–46.30)	ns
**Platelets** (×10^3^ cells/µL)	1008.14 ± 88.13	(855.00–1127.00)	1024.43 ± 110.32	(861.00–1220.00)	958.00 ± 211.00	(588.00–1161.00)	1502.83 ± 300.46	(1102.00–1920.00)	< 0.001
**Total neutrophils** (×10^3^ cells/µL)	0.48 ± 0.18	(0.18–0.66)	0.64 ± 0.39	(0.21–1.44)	0.39 ± 0.15	(0.21–0.58)	9.99 ± 4.77	(4.10–16.14)	< 0.0001
**Total lymphocytes** (×10^3^ cells/µL)	0.19 ± 0.06	(0.14–0.31)	0.22 ± 0.12	(0.07–0.43)	0.14 ± 0.05	(0.07–0.21)	0.64 ± 0.17	(0.37–0.84)	< 0.0001
**Total basophils** (×10^3^ cells/µL)	0.00 ± 0.00	(0.00–0.01)	0.00 ± 0.01	(0.00–0.01)	0.00 ± 0.00	(0.00–0.01)	0.03 ± 0.02	(0.01–0.07)	< 0.001
**Total eosinophils** (×10^3^ cells/µL)	0.08 ± 0.05	(0.04–0.17)	0.14 ± 0.15	(0.04–0.46)	0.07 ± 0.03	(0.03–0.10)	0.31 ± 0.10	(0.19–0.46)	< 0.05 *, < 0.01
**Total monocytes** (×10^3^ cells/µL)	0.06 ± 0.02	(0.04–0.09)	0.08 ± 0.18	(0.03–0.18)	0.06 ± 0.03	(0.02–0.12)	0.18 ± 0.10	(0.07–0.31)	< 0.05 ^#^, < 0.01

Table shows mean and standard deviation (± SD) of different hematological parameters in PBS-, γ-irradiated *M. brumae-*, *M. brumae-* and BCG-IV-infected mice. Abbreviations: ^a^ WBCB (White Blood Cell Count); ^b^ RCB (Red Blood Cell Count). Data are expressed as mean ± SD and the range of each parameter in mouse experimental groups. N = 7 except for indicated parameters of experimental groups that had insufficient volumes of sample: ^1^ n = 6. Significant differences were established by one-way ANOVA comparing the experimental groups versus BCG except: * (γ-irradiated- *M. brumae*, *M. brumae* vs. BCG) and ^#^ (PBS vs. BCG).

**Table 3:** Assessment of biochemical parameters in IB-inoculated BALB/c mice.

Treatment	PBS		γ-Irradiated *M. brumae* (Low Dose)		γ-Irradiated *M. brumae* (High Dose)		*M. brumae* (Low Dose)		*M. brumae* (High Dose)		*p* Value
Parameters	Mean ± SD	Range	Mean ± SD	Range	Mean ± SD	Range	Mean ± SD	Range	Mean ± SD	Range	
**ALT** (U/L) ^a^	12.80 ± 3.60 ^2^	(9.30–19.40)	16.66 ± 5.10 ^3^	(11.60–25.10)	13.08 ± 2.58 ^2^	(10.04–16.50)	13.76 ± 6.43 ^3^	(9.00–23.70)	14.87 ± 1.71 ^2^	(12.60–17.70)	ns
**AST** (U/L) ^b^	59.33 ± 18.33 ^2^	(38.00–90.00)	75.60 ± 60.60 ^3^	(42.00–183.00)	65.00 ± 16.70 ^2^	(45.00–94.00)	67.20 ± 26.00 ^3^	(51.00–113.00)	82.71 ± 28.90 ^1^	(45.00–94.00)	ns
**Creatinine** (mg/dL)	0.25 ± 0.03	(0.20–0.29)	0.28 ± 0.02 ^2^	(0.26–0.30)	0.24 ± 0.06	(0.10–0.30)	0.27 ± 0.03 ^1^	(0.25–0.33)	0.26 ± 0.02	(0.10–0.30)	ns
**Iron** (µg/dL)	145.90 ± 50.27 ^1^	(67.30–207.10)	160.26 ± 61.10 ^3^	(113.80–259.30)	133.06 ± 31.60 ^1^	(70.90–159.70)	170.67 ± 21.17 ^1^	(134.70–219.50)	165.07 ± 45.90 ^1^	(70.90–159.70)	ns
**Alkaline Phosphatase** (mmol/L)	141.87 ± 12.71 ^1^	(123.73–157.00)	138.02 ± 14.40^3^	(112.80–148.65)	147.95 ± 11.40 ^2^	(132.30–163.40)	146.74 ± 19.76 ^1^	(133.80–174.33)	149.11 ± 15.95 ^1^	(132.30–163.40)	ns
**Glucose** (mg/mL)	247.75 ± 35.92	(207.70–297.60)	287.95 ± 60.01 ^2^	(179.90–351.20)	256.63 ± 30.90 ^1^	(224.00–294.20)	256.63 ± 30.93 ^1^	(229.30–322.30)	257.23 ± 49.80 ^2^	(224.00–294.20)	ns
**Potassium** (mmol/L)	4.76 ± 0.63	(3.95–6.01)	5.04 ± 0.92 ^2^	(4.07–5.21)	4.90 ± 0.57 ^1^	(4.00–5.74)	4.90 ± 0.57 ^1^	(3.90–5.65)	4.69 ± 0.34 ^2^	(4.00–5.74)	ns
**Sodium** (mmol/L)	145.58 ± 1.97	(142.80–149.30)	144.25 ± 1.58 ^2^	(142.50–146.80)	144.66 ± 1.58 ^1^	(143.50–148.00)	144.66 ± 1.58 ^1^	(141.10–153.10)	146.90 ± 1.59 ^2^	(143.50–148.00)	ns
**Urea** (mg/dL)	40.86 ± 5.94	(32.40–50.60)	39.63 ± 9.60 ^2^	(30.10–54.80)	39.06 ± 7.36	(29.30–52.10)	39.06 ± 7.38 ^1^	(28.20–60.90)	47.56 ± 9.59 ^1^	(29.30–52.10)	ns
**Albumin** (g/dL)	2.59 ± 0.16 ^1^	(2.30–2.75)	2.65 ± 0.04 ^3^	(2.30–2.75)	2.65 ± 0.04 ^3^	(2.60–2.71)	2.65 ± 0.04 ^1^	(2.33–2.61)	2.64 ± 0.06	(2.60–2.71)	ns
**Total Protein** (g/dL)	4.54 ± 0.24 ^1^	(4.15–4.89)	4.74 ± 0.34 ^3^	(3.99–4.93)	4.74 ± 0.34 ^2^	(4.44–5.40)	4.74 ± 0.34 ^1^	(4.08–5.02)	4.60 ± 0.44	(4.44–5.40)	ns

Table shows mean and standard deviation (± SD) of different biochemical parameters in PBS-, γ-irradiated *M. brumae* (low and high doses) and *M. brumae* (low and high doses) IB-treated mice. Abbreviations: ^a^ ALT (Alanine transaminase); ^b^ AST (Aspartate transaminase). Data are expressed as mean ± SD and the range of each parameter in mouse experimental groups. N = 8 except for indicated parameters of experimental groups that had insufficient volumes of sample: ^1^ n = 7, ^2^ n = 6, ^3^ n = 5). Significant differences were established by one-way ANOVA comparing the experimental groups versus PBS.

**Table 4:** Assessment of hematology parameters in IB-inoculated BALB/c mice.

Treatment	PBS		γ-Irradiated *M. Brumae* (Low Dose)		γ-Irradiated *M. Brumae* (High Dose)		*M. Brumae* (Low Dose) ^1^		*M. Brumae* (High Dose) ^2^		*p* Value
Parameters	Mean ± SD	Range	Mean ± SD	Range	Mean ± SD	Range	Mean ± SD	Range	Mean ± SD	Range	
**WBCB**^a^ (×10^3^ cells/µL)	6.41 ± 1.58	(4.05–8.42)	6.59 ± 1.78	(4.78–9.28)	5.53 ± 1.35	(3.57–7.37)	4.40 ± 1.01	(2.7–5.28)	4.79 ± 1.51	(3.06–6.12)	ns
**RCB**^b^ (×10^3^ cells/µL)	9.34 ± 0.48	(8.60–9.87)	9.13 ± 0.41	(8.79–9.65)	9.62 ± 0.43	(9.02–10.15)	9.01 ± 0.34	(8.65–9.43)	9.12 ± 0.34	(8.68–9.42)	ns
**Hematocrit** (%)	41.07 ± 1.60	(39.40–43.30)	41.20 ± 2.33	(38.50–45.40)	42.3 ± 1.55	(40.90–44.40)	40.42 ± 1.82	(37.9–43)	40.17 ± 0.67	(39.30–40.90)	ns
**Platelets** (×10^3^ cells/µL)	953.00 ± 103.48	(809.00–1076.00)	929.00 ± 150.04	(656.00–1073.00)	859.33 ± 253.51	(388.00–1155.00)	836.00 ± 265.49	(367.00–996.00)	854.00 ± 179.37	(595.00–989.00)	ns
**Total neutrophils** (×10^3^ cells/µL)	0.94 ± 0.38	(0.47–1.49)	0.98 ± 0.25	(0.70–1.39)	0.76 ± 0.29	(0.31–1.10)	0.53 ± 0.14	(0.39–0.68)	0.66 ± 0.26	(0.39–1.01)	ns
**Total lymphocytes** (×10^3^ cells/µL)	4.97 ± 1.30	(3.05–6.27)	5.21 ± 1.40	(3.64–7.17)	4.35 ± 0.98	(2.80–5.07)	3.53 ± 0.93	(2.05–4.59)	3.56 ± 1.25	(2.47–4.83)	ns
**Total basophils** (×10^3^ cells/µL)	0.01 ± 0.01	(0.00–0.01)	0.01 ± 0.01	(0.01–0.02)	0.01 ± 0.01	(0.00–0.03)	0.01 ± 0.01	(0.00–0.01)	0.01 ± 0.01	(0.00 – 0.01)	ns
**Total eosinophils** (×10^3^ cells/µL)	0.17 ± 0.11	(0.07–0.37)	0.14 ± 0.04	(0.09–0.22)	0.15 ± 0.02	(0.12–0.18)	0.13 ± 0.02	(0.11–0.15)	0.33 ± 0.30	(0.11–0.76)	ns
**Total monocytes** (×10^3^ cells/µL)	0.13 ± 0.09	(0.06–0.32)	0.11 ± 0.03	(0.06–0.16)	0.11 ± 0.04	(0.09–0.20)	0.05 ± 0.01	(0.09–0.06)	0.08 ± 0.04	(0.06–0.15)	ns

Table shows mean and standard deviation (± SD) of different biochemical parameters in PBS-, γ-irradiated *M. brumae-* (low and high doses) and *M. brumae-* (low and high doses) IB-treated mice. Abbreviations: ^a^ WBCB (White Blood Cell Count); ^b^ RCB (Red Blood Cell Count). N = 6 except for indicated parameters of experimental groups that had insufficient volumes of sample: ^1^ n = 5, ^2^ n = 4. Data are expressed as mean ± SD and the range of each parameter in mouse experimental groups. Significant differences were established by one-way ANOVA comparing the experimental groups versus PBS.

## Supplementary Materials

The following are available online at https://www.mdpi.com/2076-393X/8/2/198/s1, Figure S1: Body weight and relative body weight of mice annotated along the study, Figure S2: Bacterial burden from IB-treated mice.

## Appendix A

Mycobacterium genera has recently divided in four different new genera. *Mycobacterium brumae* is officially denominated *Mycolicibacterium brumae*.
