# Supplementary material for: Mycolicibacterium brumae is a Safe and Non-Toxic Immunomodulatory Agent for Cancer Treatment
Source: Vaccines (Basel). 2020 Apr 25;8(2):198. doi: 10.3390/vaccines8020198 (PMC7349652; doi:10.3390/vaccines8020198)
Supplement: Supplementary file 1 [file vaccines-08-00198-s001.pdf]

vaccines

MDPI

Article

Mycolicibacterium brumae is a Safe and Non-Toxic Immunomodulatory Agent for Cancer Treatment

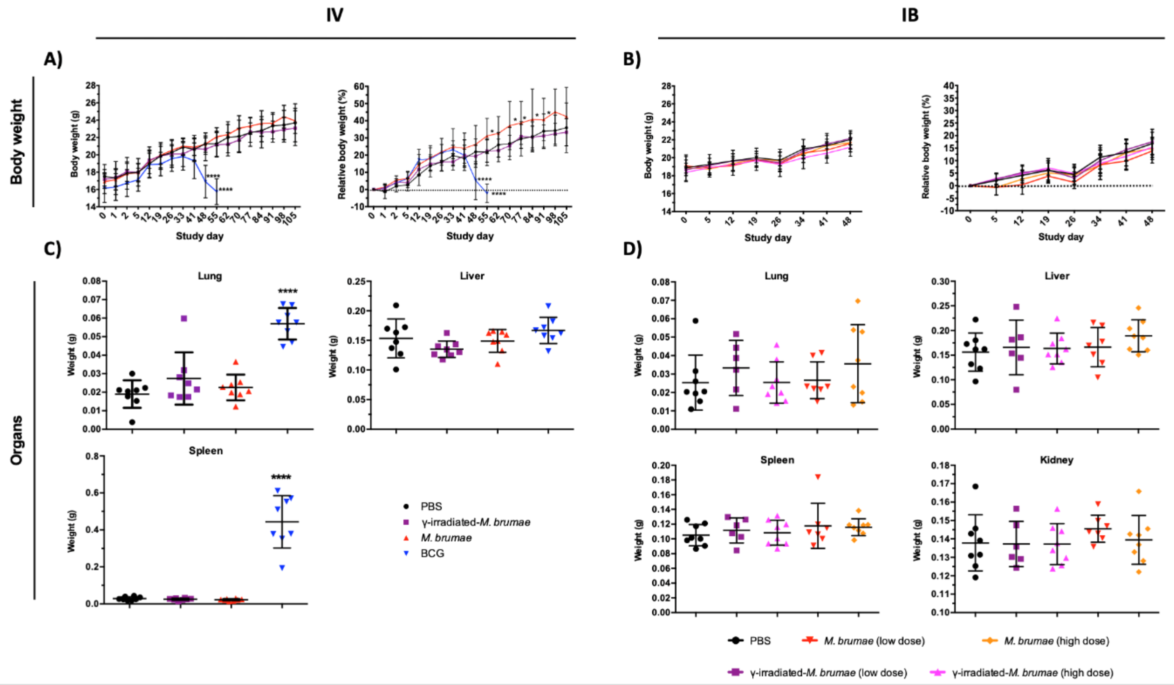

Figure 1. Body weight and relative body weight of mice annotated along the study in (**A**) IV-infected SCID mice and (**B**) IB-treatment BALB/c mice. * $p < 0.05$; *** $p < 0.001$ (One-way ANOVA). Organ weights of the (**C**) lungs, livers and spleens from IV-infected SCID mice and (**D**) lungs, livers, spleens and kidneys from IB-treated BALB/c mice. Data are presented as the mean values ± SD. **** $p < 0.0001$ (One-way ANOVA).

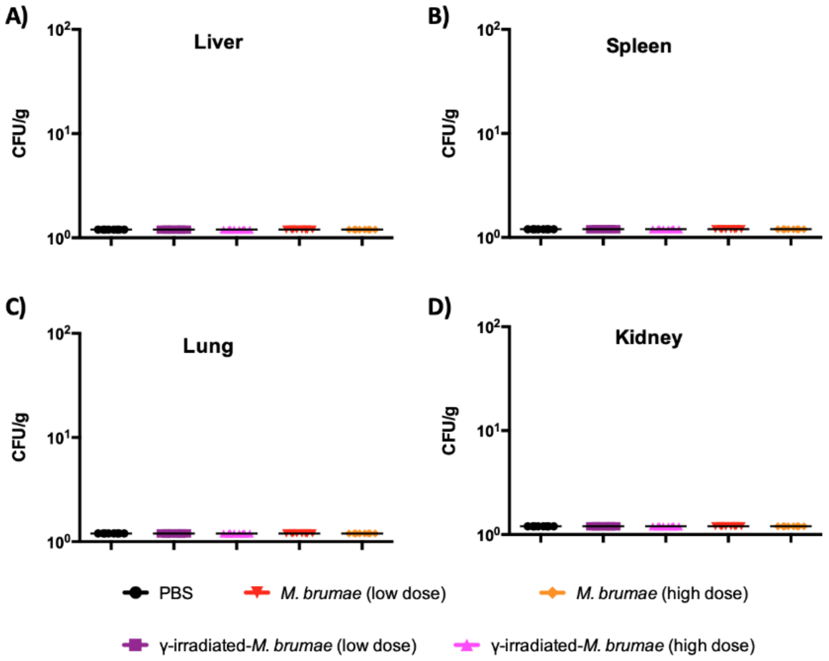

Figure 2. Bacterial burden from IB-inoculated mice. No CFU were detected in (**A**) liver, (**B**) spleen, (**C**) lungs, or (**D**) kidneys following IB-inoculation of low and high doses of live or γ-irradiated *M. brumae*. Data are presented as the mean values ± SD. No-significant differences were seen (Kruskal–Wallis H test).

© 2020 by the authors. Submitted for possible open access publication under the terms and conditions of the Creative Commons Attribution (CC BY) license (http://creativecommons.org/licenses/by/4.0/).
